# Prototype development of an electrical impedance based simultaneous respiratory and cardiac monitoring system for gated radiotherapy

**DOI:** 10.1186/1475-925X-13-144

**Published:** 2014-10-14

**Authors:** Kirpal Kohli, Jeff Liu, Devin Schellenberg, Anand Karvat, Ash Parameswaran, Parvind Grewal, Steven Thomas

**Affiliations:** Department of Medical Physics, Fraser Valley Center, BC Cancer Agency, 13750 96th Avenue, Surrey, V3V 1Z2 BC Canada; Department of Radiation Oncology, Fraser Valley Center, BC Cancer Agency, 13750 96th Avenue, Surrey, V3V 1Z2 BC Canada; School of Engineering Science, Simon Fraser University, Burnaby, V5A1S6 BC Canada

**Keywords:** Bioimpedance, Respiratory and cardiac monitoring, Gated radiotherapy, Electrode positions, Signal-to-noise ratio

## Abstract

**Background:**

In radiotherapy, temporary translocations of the internal organs and tumor induced by respiratory and cardiac activities can undesirably lead to significantly lower radiation dose on the targeted tumor but more harmful radiation on surrounding healthy tissues. Respiratory and cardiac gated radiotherapy offers a potential solution for the treatment of tumors located in the upper thorax. The present study focuses on the design and development of simultaneous acquisition of respiratory and cardiac signal using electrical impedance technology for use in dual gated radiotherapy.

**Methods:**

An electronic circuitry was developed for monitoring the bio-impedance change due to respiratory and cardiac motions and extracting the cardiogenic ECG signal. The system was analyzed in terms of reliability of signal acquisition, time delay, and functionality in a high energy radiation environment. The resulting signal of the system developed was also compared with the output of the commercially available Real-time Position Management™ (RPM) system in both time and frequency domains.

**Results:**

The results demonstrate that the bioimpedance-based method can potentially provide reliable tracking of respiratory and cardiac motion in humans, alternative to currently available methods. When compared with the RPM system, the impedance-based system developed in the present study shows similar output pattern but different sensitivities in monitoring different respiratory rates. The tracking of cardiac motion was more susceptible to interference from other sources than respiratory motion but also provided synchronous output compared with the ECG signal extracted. The proposed hardware-based implementation was observed to have a worst-case time delay of approximately 33 ms for respiratory monitoring and 45 ms for cardiac monitoring. No significant effect on the functionality of the system was observed when it was tested in a radiation environment with the electrode lead wires directly exposed to high-energy X-Rays.

**Conclusion:**

The developed system capable of rendering quality signals for tracking both respiratory and cardiac motions can potentially provide a solution for simultaneous dual-gated radiotherapy.

## Background

Radiotherapy of tumors in the thoracic region is affected by motion of organs due to respiration and cardiac contractions. Respiratory-gated radiotherapy offers a significant potential for improvement in the irradiation of tumour sites affected by respiratory motion such as lung, breast, pancreas and liver tumours. Respiratory gating of radiation therapy involves limiting delivery of radiation to optimum parts of the respiratory cycle. The position and width of the gate within a respiratory cycle are determined by monitoring the patient’s respiratory motion, using either an external respiration signal or internal fiducial markers [1–6]. Currently one example of a commercially available respiratory gating system is the Real-time Position Management™ (RPM) system (Varian Medical Systems, Palo Alto, CA). In this system an external marker device is placed on the abdomen between the xiphoid process and the umbilicus. An infrared camera tracks the motion of the marker, and that motion generates a surrogate for the respiratory cycle. Respiratory position monitoring has been extremely valuable to radiation oncology but is not ideal for all patients and suffers from a few short comings [7]. The marker block is difficult to position in patients with certain body habitus and is poorly mobile in certain patients who do not breathe with their diaphragm while patients with poor lung function have little chest/abdominal wall excursion so the block does not move and a respiratory tracing cannot be obtained. Furthermore, there could be an inherent lag between external marker motion and internal anatomy motion [8, 9]. Finally, the RPM system has no means of detecting cardiac motion that could alter tumor and/or organ at risk position.

Another internal marker based gating system is a miniature, implantable powered radiofrequency (RF) coil that can be tracked electromagnetically in three dimensions from outside the patient [4]. The performance of a wireless RF seed tracking system for tumor localization has been reported [1]. Even though this system is considered to be accurate, it involves an invasive procedure and there are minor risks associated with this, including pain, bleeding, and infection. In addition, tracking a few localized internal markers does not comprehensively account for the motion of the many normal structures adjacent to tumors. Furthermore, though beacon transponders are safe for magnetic resonance imaging (MRI) they produce a local image artifact in adjacent tissue [10].

The respiration process consists of rhythmic ventilation of the lungs governed by the expansion and contraction of the chest cavity, which is controlled by the intercostal muscles and the diaphragm. The periodic physiological change of the chest cavity, in turn, results in a rhythmic change in bio-impedance. Ohm’s Law depicts that for an electrically conductive object, the current flow is directly proportional to the applied electrical potential and the ratio of the electrical potential to the current is the electrical impedance of the object. The linearity of the current–voltage relationship also implies that if the current is kept constant, the change in the voltage is proportional to the change in impedance.

Cardiac motion has received less attention due to a lower magnitude of motion as compared to respiration as well as the relative infrequency of cardiac tumors as compared with lung [11–14]. Although physical displacement is less for cardiac motion than it is for respiration, a displacement of up to 8 mm due to cardiac motion has been shown [13] which is significant for high precision radiation therapy. Furthermore, with increased interest in high dose per fraction treatment and the treatment of centrally located lung tumors [15], understanding cardiac motion is becoming of greater importance [16] (RTOG 0813).

In the current study we develop a prototype electrical impedance based system for simultaneous recording of respiratory and cardiac motion which will allow for both respiratory and cardiac gating simultaneously. The principle of this technique is based on the change in trans-thoracic impedance (ΔZ) that is measured through the respiratory and cardiac cycles. The bio-impedance (Z) is defined in terms of an instantaneous ratio of voltage (V) over current (I). These quantities can be easily measured non-invasively in real time by transmitting a known low-amplitude and low-frequency current (I) and measuring voltage drop (ΔV) across electrodes attached to the thorax. Numerous studies have shown that change in trans-thoracic impedance as a function of time can be correlated with breathing [17, 18].

In humans, an inspiration maneuver from residual volume to total lung capacity results in a regional bio-impedance change of 300% [19]. Cardiac activity and perfusion also cause a change in thoracic bio-impedance, from diastole to systole, in the range of 3% [20]. Since respiratory and cardiac functions can be monitored using similar methods of bio-impedance measurements, this technique offers a novel and truly non-invasive method for a dual gating system which can be accomplished by a single sensor or device. As demonstrated previously [21, 22], a strong correlation can be achieved between signals acquired through bio-impedance and the conventional methods of respiratory analysis (pneumotachography) and cardiac analysis (ECG). In Koivumaki’s device the raw bio-impedance based electrical signal was first sampled and then respiratory and cardiac components were extracted based on different frequencies using software. Unfortunately, extra delay was introduced after the sampling of the data due to data processing. Our study, on the other hand, aims to separate the respiratory and cardiac signals by hardware means using electronic circuits, prior to the signal sampling step. Such an approach has the advantage of effectively reducing the time delay caused by the post-sampling data processing. The aim of our study is to provide a real-time gating signal for radiation therapy, in which case the time delay between the actual physiological change and the rendering of the signal should be minimized, by largely reducing the time required for post-sampling data processing. Furthermore, in the previous study conducted [21, 22], the direct current (DC) component of the sampled signal was filtered out in order to remove baseline fluctuation of the respiratory signal. However, in events such as momentary breath holding, the filtered sampled signal cannot correctly represent the position of the chest cavity, which will result in error if the signal is to be used for gating the actual treatment. Our study develops an algorithm for mitigating this issue by ignoring temporary DC level shifts.

In this work a prototype bio-impedance system is designed, custom built and evaluated for the application of real-time simultaneous respiratory and cardiac gated radiotherapy.

## Materials and methods

### Acquisition of impedance-based respiratory monitoring signal

**Figure 1:**
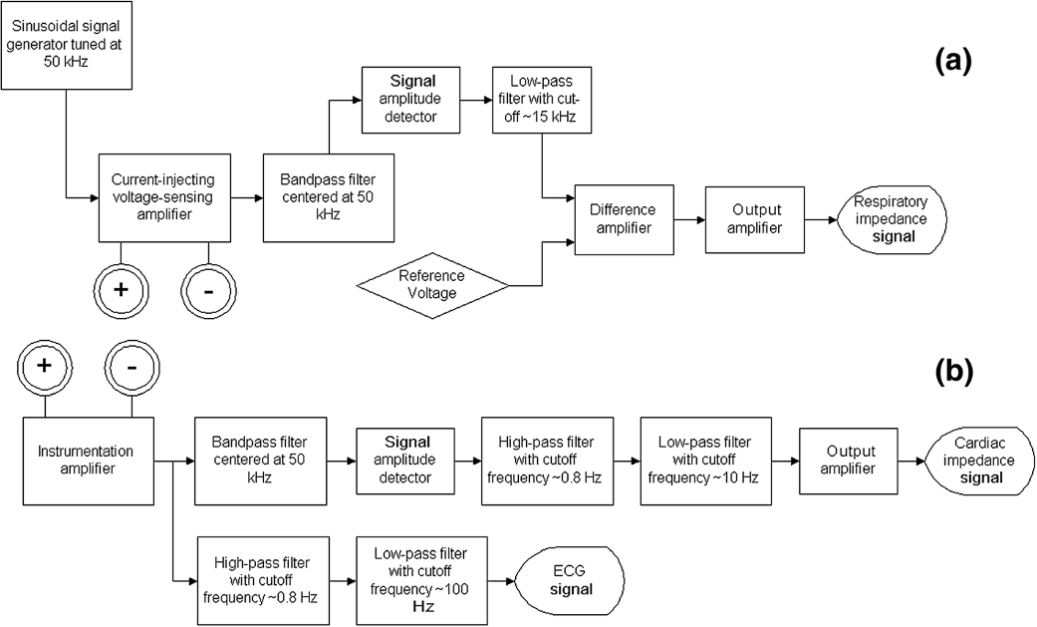
**Block diagrams of (a) the respiratory motion monitoring circuit and (b) the cardiac motion monitoring circuit developed.** This cardiac monitoring circuit has to be operated in conjunction with the respiratory monitoring unit, because the cardiac monitoring circuit does not include a carrier signal source.

The impedance based respiratory monitoring system mainly consists of two modules, the current-injecting module and the voltage measuring module. Figure 1a shows the block diagram of the system built on a single circuit board. The current-injecting module is capable of sourcing and sinking current based on the waveform of the input signal to the module, irrespective of the magnitude of the impedance. The source signal generated has a pre-defined amplitude and frequency that can be identified and isolated by the voltage-measuring module of the detection circuit. The voltage-measuring module specifically calibrated for the carrier signal also provides the required amplification so the output of the detection circuit can be properly sampled and analyzed.

In the present implementation, the source signal of the current-injecting module is generated using an UG-268 evaluation board for AD9838 direct digital synthesizer configured by a Blackfin® (SDP-B) controller board manufactured by Analog device (Analog Devices, Norwood, Massachusetts, USA). Meanwhile, the current-injecting voltage-sensing amplifier, signal amplitude detector, low-pass filter, difference amplifier, and output amplifier are all constructed using TL074 operation amplifiers. The signal detecting unit is designed to monitor the variation in the resistive component of the measured bio-impedance. It is therefore the amplitude information of the carrier signal that is extracted for tracking breathing motion. In order to achieve high sensitivity for the correlation between the recorded signal and breathing motion, a sharp, high-quality 8th-order bandpass filter with a center frequency the same as the carrier signal frequency is used for screening out unwanted noise. Such a bandpass filter is constructed using a commercially available LTC®1264 universal filter block chip (Linear Technology, Milpitas, California, USA). An 8th-order bandpass filter (created by cascading four 2nd-order filter blocks) amplifies signal components within the frequency band centered at the carrier frequency while largely attenuates components outside the passband frequency.

The bioimpedance measurement on the three human subjects was performed with the current-injecting and current-sinking electrode pair placed laterally across the thorax. Specifically, the electrodes were placed approximately 2–3 cm inferior to the axillary fold in the mid-axillary line on both the right and left chest.

### Acquisition of impedance-based cardiac monitoring signal

Bio-impedance changes due to cardiac motion can be simultaneously measured using the same carrier signal employed for respiratory monitoring by placing a second pair of electrodes on the thorax in close proximity of the heart (the first placed along the sternum, at the level of 6th sternocostal junction, and the 2nd 4 cm lateral to the first electrode on the left side), aligned with the above-mentioned electrode pair for respiratory monitoring. This pair of cardiac electrodes does not source or sink any electrical current and thus causes insignificant (if any) interference with the carrier signal injected through the current sourcing/sinking pair of electrodes. Such configuration of electrodes is similar to the 4-point probe method in electrical measurement. In order not to sink any significant carrier signal, the pair of electrodes for sensing the bio-impedance due to the cardiac cycle must have very large input impedance. Figure 1b shows the circuit implemented for monitoring cardiac induced bio-impedance change in the present study. The pair of electrodes for cardiac monitoring is connected to the input of an instrumentation amplifier implemented using a commercially available AD620 chip (Analog Devices, Norwood, Massachusetts, USA). The instrumentation amplifier measures the differential voltage across the electrode pair while providing very large input impedance. Following the input instrumentation amplifier is a bandpass filter implemented using LTC®1264 universal filter block chip, with a center frequency the same as the carrier signal frequency and a signal envelope detecting block to extract the amplitude information contained in the carrier signal. The high-pass filter in the cardiac impedance monitoring circuit is an 8th-order filter constructed using a LTC®1068 quad 2nd-order filter block chip by Analog Devices. A sharp cut-off at the corner frequency of the high-pass filter is necessary to remove the signal components induced by respiration in the cardiac signal. Meanwhile, the low-pass filtering in the cardiac impedance monitoring circuit is implemented using Sallen-Key configuration of TL074 op-amps.

The discrepancy between the acquisition of the respiratory gating signal and that of the cardiac gating signal is the pass band frequency of the output filter. To extract the respiratory impedance signal, the output filter consists of a low-pass filter with a cutoff frequency at approximately 15 Hz. Because of the locations at which the respiratory motion sensing electrodes are placed, the variation of the signal due to the cardiac activity is negligible compared to that due to the respiratory activity. However, on the other hand, for the cardiac motion sensing electrodes, even though these electrodes are located close to the heart, the interference due to respiration in the signal sensed is sufficiently large to distort the shape of the cardiac impedance signal. To provide a clean cardiac bio-impedance signal for gating purpose, the variation contained in the sensed signal due to respiratory motion has to be removed. A sharp signal filter is used to subtract the respiratory component out of the cardiac signal. At rest, normal human respiratory activity usually results in a signal frequency below 0.5 Hz (30 breaths/min). Meanwhile, normal human heart rate is almost always above 1 Hz (60 beats/min). The interference due to respiratory motion is largely removed by a sharp high-pass filter with a cutoff frequency of 0.8 Hz. Furthermore, a low-pass filter with a cutoff frequency of 10 Hz is used to remove high frequency noise.

**Figure 2:**
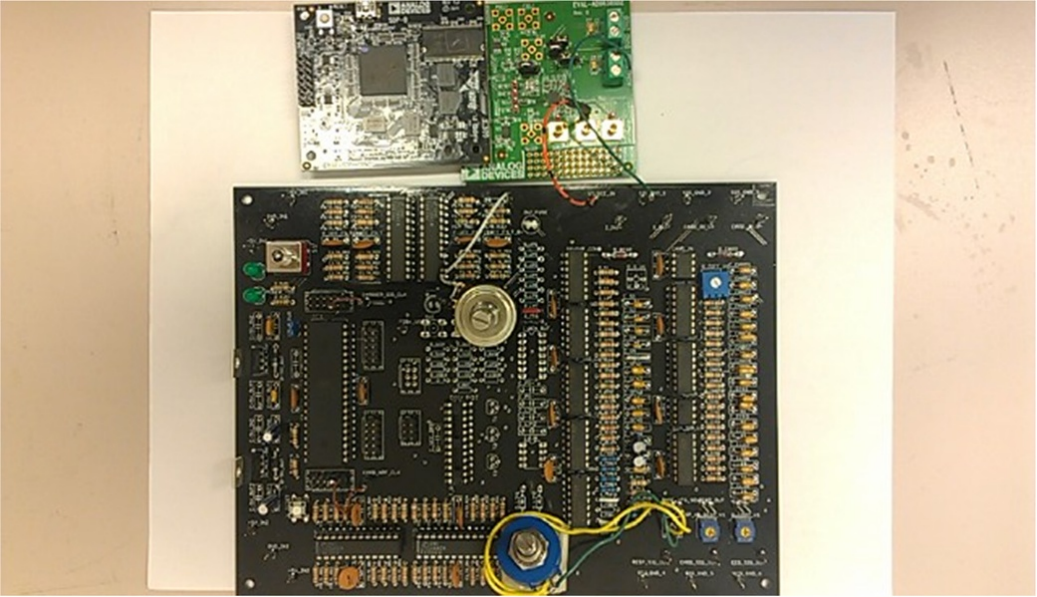
**A snapshot of the respiratory and cardiac impedance monitoring and cardiogenic signal extracting circuit implemented on a printed circuit board.**

To compare with the cardiac induced bio-impedance signal recorded, the cardiogenic signal was also acquired. The same cardiogenic signal is commonly used in electrocardiography (ECG). The acquisition of the ECG signal in the present design is through the same electrode pair that monitors the bio-impedance change due to the cardiac motion. However, instead of observing the variation of the 50 kHz carrier signal, the circuit only amplifies signal with frequencies between 0.8 Hz and 100 Hz but suppresses signal outside this frequency region. The selection of such frequency band allows the carrier signal to be effectively excluded and the electrical potential generated by the sinoatrial node in the heart to be observed. Such electrical potential consists of mostly frequency components less than 100 Hz. By using the same pair of electrodes that sense the cardiac-induced bio-impedance change, no additional electrodes are required for acquiring the ECG signal. The schematic of ECG signal extracting circuit is also illustrated in Figure 1b. A snapshot of the entire circuit on a printed circuit board in operation is shown in Figure 2.

### Determining time delay using electronic simulator

In order to characterize the time delay between the change of the actual resistance under test and the change of signal at the output of the respiratory monitoring circuitry developed, a variable resistance circuit consisting of a digital potentiometer (AD5206, Analog Devices Norwood, Massachusetts, USA) controlled by a microcontroller programmed to output a pre-defined waveform was used. The current-injecting and current-sinking leads of the respiratory motion monitoring circuit were connected on the two terminals of the digital potentiometer.

The microcontroller was programmed to control a digital potentiometer with resistance changing according to a periodic rectangular pulse of 2.5 kΩ amplitude. In a single period of 2.79 s the resistance was such that the potentiometer continuously stayed at 0.4 kΩ but periodically increased its value to 2.5 kΩ for a short duration of approximately 11 ms and then dropped back to 0.4 kΩ. Such pattern of resistance change created a short rectangular pulse function that allowed the monitoring of time delay. With the assumption that the propagation delay between the actual change of the resistance experienced and the output of the signal injecting amplifier is negligible, the propagation delay of the respiratory monitoring circuit can be characterized by comparing the output of the current-injecting amplifier and the final output of the respiratory monitoring circuit. For our measurements; the delay is defined by the time from the center of the higher resistance portion of the square wave to the highest value of the detector circuit output.

To determine the propagation delay of the cardiac monitoring circuit, a similar testing circuit was set up using the AD5206 digital potentiometer controlled by a microcontroller. However, since the cardiac induced bio-impedance measurement employs a 4-point probe technique, two 5-kΩ fixed value resistors were connected on the two terminals of the digital potentiometer. Signal was injected and collected from the two ends of the 5-kΩ resistors, while the differential voltage across the digital potentiometer was measured. The digital potentiometer was programmed to follow a periodic rectangular pulse function with 0.78 s period and 6 ms pulse duration. If the internal delay of the input instrumentation amplifier is assumed to be insignificant, the propagation delay of the cardiac monitoring circuit can be determined by comparing the output signal of the instrumentation amplifier with the final output signal of the cardiac monitoring circuit.

### Functionality test in a radiation environment

Because the objective for the present study is to develop a respiratory and cardiac monitoring system that can be eventually applied to gated radiotherapy, correct functioning of the electronic device in a radiation environment is critical. To test the electronics the prototype system was placed in the vicinity of a high-energy X-Ray beam. The effect of radiation on the system was tested using the variable resistance circuit mentioned above. The digital potentiometer was programmed to output a sinusoidal resistance with a period of approximately 3 seconds to simulate breathing motion. A pair of lead wires embedded in a 50-cm coaxial cable connected the output terminals of the current sourcing amplifier across the variable resistance circuit. The lead wires were placed inside the radiation field and directly irradiated with a 6 MV beam to ~100 cGy dose at 400 MU/min and the field size was 10×10 cm^2^ while the respiratory monitoring circuit and the variable resistance circuit were located outside the radiation field. The output of the respiratory monitoring circuit was recorded with and without the high-energy beam switched on. In the first trial, the variable resistance was left running according to the input sinusoidal waveform for 120 seconds without irradiation. In the second trial, the X-Ray beam was switched on 60 seconds after recording started and turned off when the total dose reached 100 cGy. Results of the two test conditions were compared.

## Results

### Respiratory monitoring

**Figure 3:**
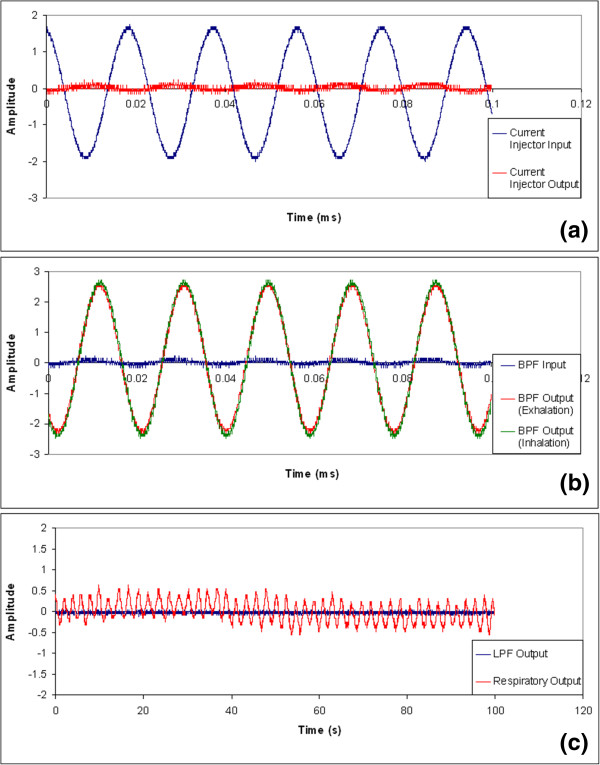
**Observed signals at (a) the input and output of the current injector, (b) the input and output of the bandpass filter (BPF) during inhalation and exhalation, and (c) the output of the low-pass filter (LPF) and the output of the respiratory monitoring circuit.** The subject was a 172-cm, 75-kg, 32-year-old male.

The electrical signals observed at the input stage of the respiratory monitoring circuitry developed are illustrated in Figure 3. Figure 3a shows the input and output signals of the current injecting circuit recorded when the circuit was tested on a healthy volunteer. The input signal was a zero-centered 4 V peak-to-peak sinusoidal waveform. It can be observed that the output signal was largely attenuated and had a much smaller signal-to-noise ratio compared with the input signal, due to the electrical impedance of the human body tissues. By comparing the input and output of the current injecting circuit block a phase shift in the carrier signal can be observed. However, such a phase shift does not introduce significant time delay in respiratory monitoring because the information incorporated in the carrier signal varies at a much lower frequency compared with that of the carrier signal. Figure 3b shows the input signal of the band pass filter from the current injector and the amplified output signals of the bandpass filter recorded on the healthy volunteer during exhalation and inhalation. It is shown that the bandpass filter significantly improved the signal-to-noise ratio of the carrier signal. It can also be observed that there is a small but detectable change in the output signal amplitude between the inhaling and the exhaling phases.

**Figure 4:**
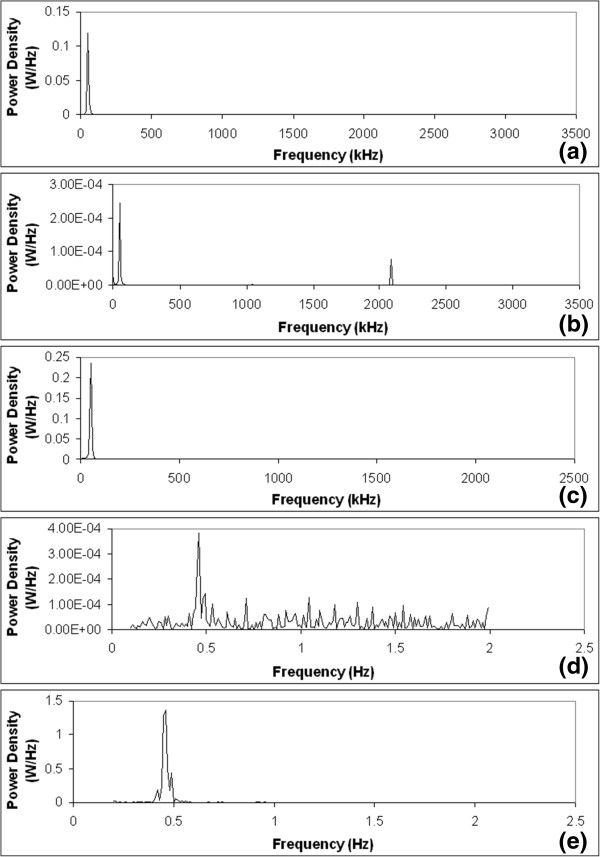
**Power spectral densities of signals at (a) current injector input, (b) current injector output, (c) bandpass filter output, (d) low-pass filter output, and (e) respiratory output.**

Following the carrier signal bandpass filter, a signal envelope detector is used for monitoring the amplitude variation of the filtered carrier signal. A signal envelope detector only extracts the amplitude information of a sinusoidal signal while discarding its frequency information. The output signal of the signal envelope detector is further conditioned by a low-pass filter prior to the subtraction of the DC voltage component and output signal amplification. The subtraction of the DC component followed by the amplification of the signal rendered an output electrical signal that traces the respiratory motion consistently. The subtraction of the DC component is to avoid saturating the output amplifier by centering the signal around the ground level. DC subtraction is more suitable than high-pass filtering in this application by tolerating temporary DC shift due to respiratory-related physiological change. A sample of simultaneous traces of the low-pass filter output and the final output of the circuit are shown in Figure 3c. The power spectral density of the intermediate signal at each stage of the implemented respiratory monitoring circuit is presented in Figure 4. The relative signal-to-noise ratio can be estimated by comparing the area under the main peak which indicates the signal of interest and the area under other discernable peaks in the tail regions on both sides of the main peak. The larger the main peak and the smaller the area in the tail regions, the better noise performance the signal is. The signal-to-noise ratios at different stages of the circuit implemented are shown in Table 1. In the stages prior to the signal envelope detector, the signal of interest is the carrier signal, and it is desirable to have a large peak located at 50 kHz while the other regions in the spectrum being suppressed. At stages following the low-pass filter, the signal of interest would be located at approximately 0.2-0.5 Hz for normal breathing rate. A sufficiently high SNR was achieved at the output stage of the respiratory monitoring unit.

**Table 1:** **Estimated signal-to-noise ratio of signals at different stages of the circuitry implemented**

	Injected signal	Current injector output	Band-pass filter output (Exhale)	Band-pass filter output (Inhale)	Low-pass filter output	Respiratory output
Signal-to-noise Ratio	19.63	1.19	28.33	28.61	0.02	3.98

### Validation against RPM system

**Figure 5:**
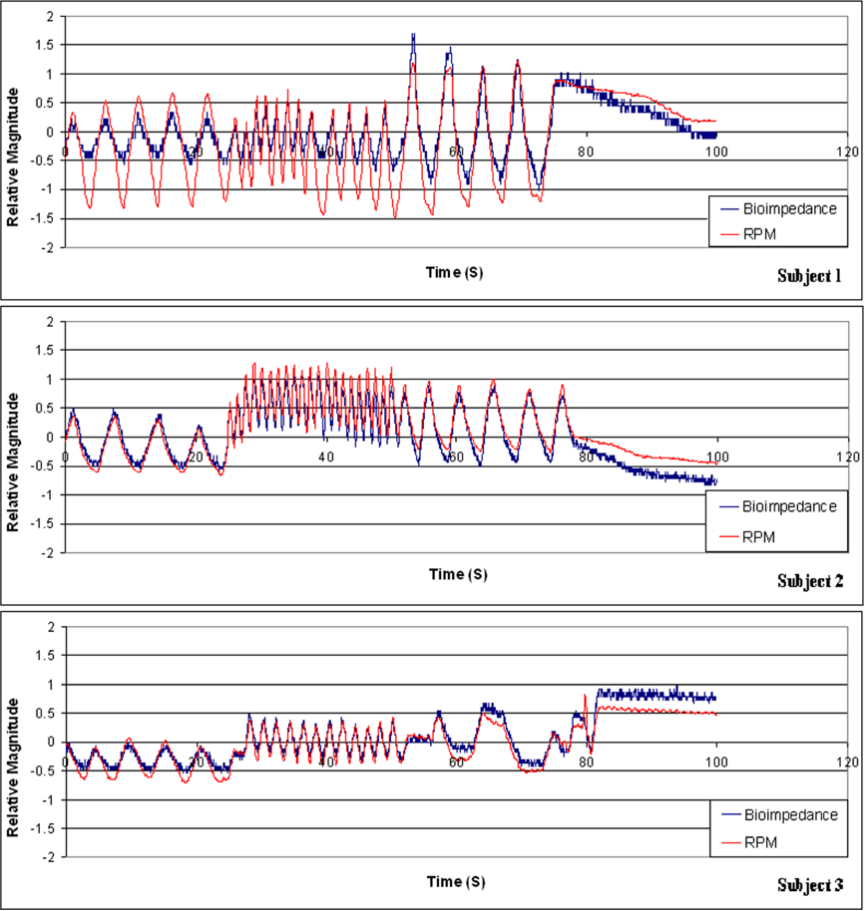
**Output traces from the bioimpedance-based respiratory monitoring unit developed and the RPM system for three healthy human volunteer.** Each subject was instructed to exercise 25 seconds of normal breathing followed by 25 seconds of fast breathing followed by 25 seconds of slow breathing and finally 25 seconds of breath holding. Subject 1: female, 168 cm, 58 kg; Subject 2: male, 183 cm, 77 kg; Subject 3: male, 177 cm, 75 kg. Correlation coefficients: Subject 1 – 0.6623; Subject 2 – 0.9502; Subject 3 – 0.9599.

**Figure 6:**
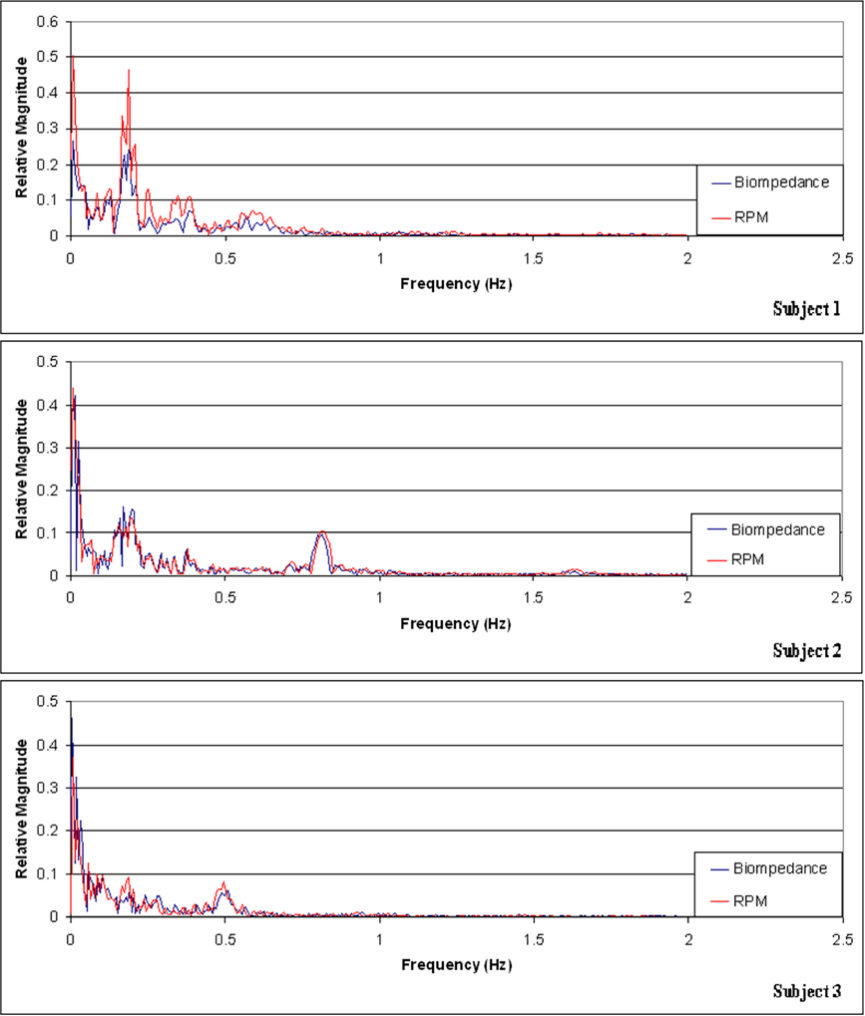
**Frequency plots of the signals shown in Figure** 5.

The bioimpedance-based respiratory monitoring unit developed was validated against the commercially available Real-time Position Management™ (RPM) system on three healthy human volunteers. Output signals were recorded using both systems simultaneously on the human subjects while each subject was instructed to follow a specific breathing pattern during the recording. The observed respiratory traces using both the bioimpedance-based unit and the RPM system for the three human subjects are shown in Figure 5. The bioimpedance-based respiratory trace was scaled and overlaid on the trace rendered by the RPM system. Both traces exhibit similar patterns following the respiratory motion in the test subjects. However, close comparison of the respiratory traces obtained by the two different monitoring mechanisms reveals that the two methods have different sensitivities in detecting respiratory cycles for different breathing rates. More specifically, the two methods render different ratios in the magnitudes of signal fluctuation during a respiratory cycle for different breathing rates. The correlation coefficients between the RPM and bio-impedance signal traces were computed to be 0.6623, 0.9502, and 0.9599 for subjects 1, 2, and 3, respectively, using the standard Matlab routine based on the correlation coefficient equation. The frequency plots of the recorded signals shown in Figure 5 are presented in Figure 6. By comparing the frequency plot of the impedance-based trace on that of the RPM trace, it can be further corroborated that the impedance-based and RPM respiratory monitoring methods have different sensitivities in detecting different respiratory rates. Notably the frequency plots also reveal that the cardiac motion did not induce significant distortion in the respiratory signal detected using the impedance-based method, as no observable peaks are present in the vicinity of 1.2 Hz in the frequency plots.

### Cardiac monitoring

**Figure 7:**
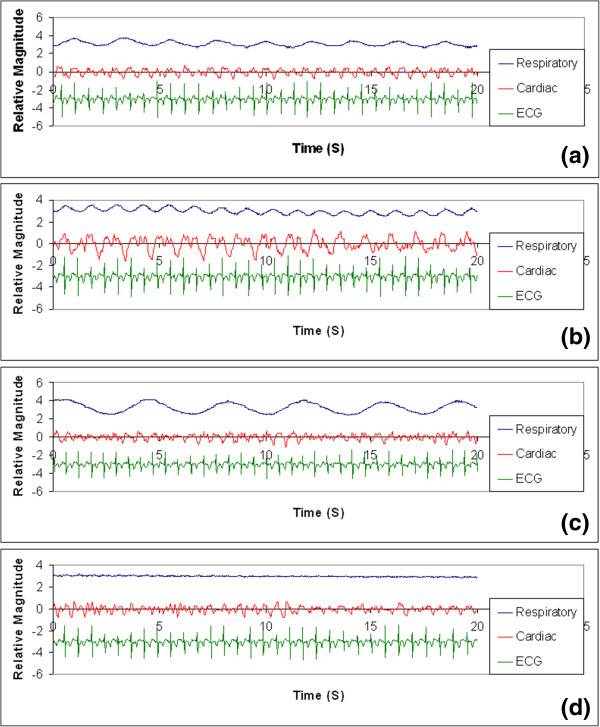
**The output waveforms of both the respiratory monitoring unit and the cardiac monitoring unit together with the ECG signal recorded during (a) normal breathing, (b) rapid breathing, (c) slow breathing, and (d) breath holding.** The subject was a 172-cm, 75-kg, 32-year-old male.

The change in impedance due to respiratory motion and cardiac motion, together with the ECG signal recorded during normal breathing pace, slow respiration, breath-holding, and fast inhalation-exhalation recorded on one of the human subjects is shown in Figure 7. We observed that the respiratory component is dominant in the unprocessed signal compared to cardiac impedance signal. It is observed that after appropriate filtration over a 20-second period, the impedance change due to cardiac motion can be more clearly traced when the test subject breathed at a normal pace, most likely due to the relaxed state of the subject. The cardiac induced bio-impedance change can be observed to vary at the same base frequency as the recorded ECG signal. However, when the subject intentionally breathed at a faster or a slower rate, the cardiac impedance signal was more distorted. It can be inferred from Figure 7 that the ECG signal sampled was more immune to the interference caused by the respiratory motion than the cardiac-induce bio-impedance signal. Impedance change due to cardiac contraction was more significantly affected when the subject breathed more rapidly than the normal rate. A cardiac gating signal that both renders more anatomical detail of the cardiac motion and is more resistant to ambient interference can potentially be provided by combining the cardiac impedance signal with the ECG signal.

### Time delay for respiratory monitoring

**Figure 8:**
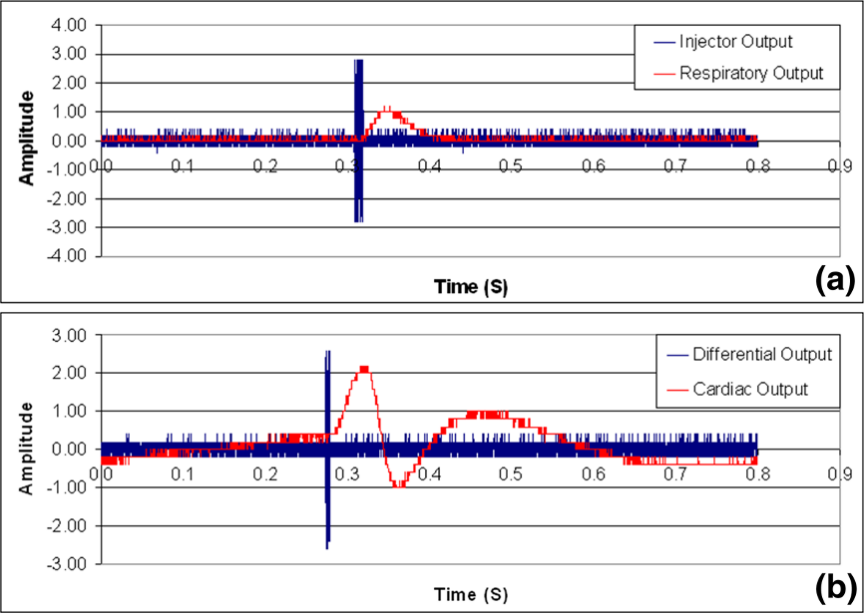
**Characterization of propagation delay of (a) respiratory monitoring circuit and (b) cardiac monitoring circuit by introducing a short pulse in the resistance under test.**

Figure 8 is a sample simultaneous display of the current-injecting amplifier output signal and the output signals of both the respiratory induced and cardiac induced bio-impedance monitoring circuits. The time delay introduced by the circuit was observed to be approximately 33 ms. The majority of time delay in the circuit was observed to be caused by the low pass filter. Such time delay results from the narrow frequency pass band of the low pass filter. A propagation delay of apporoximately 45 ms was observed for the cardiac monitoring circuit. The time delay was recorded as the time difference between the input impulse signal of these circuits and the occurrence of maximum peak at the output.

### Effect of radiation

The functionality of our monitoring circuit was evaluated in a treatment 6MV linear accelerator environment. The respiratory monitoring unit was observed to function normally in the radiation environment tested. An approximately 0.06% difference in the variance of the output signals for the two test conditions was observed. Such a difference is insignificant compared to the magnitude of monitored signal.

## Discussion

Our results demonstrate that the prototype system that we built can monitor simultaneous respiratory and cardiac motion in real time which has a potential to implement simultaneous respiratory and cardiac gated radiotherapy. Quality cardiac and respiratory signals were obtained with our current electronic circuitry for all the subjects we studied. In our prototype device we have separated the respiratory and cardiac signals by hardware means using electronic circuits, prior to the signal sampling step. Our approach has the advantage of effectively reducing the time delay caused by the post-sampling data processing and thus has the potential to enable simultaneous cardiac and respiratory gated treatment. Previous studies have suggested a breath-hold approach for separating the respiratory and cardiac signals. The breath-hold approach captures the cardiac related impedance signal without the need for filtering, but lacks the ability to measure the interactions between ventilation and cardiac signals.

In the current implementation we have used 50-kHz frequency and we believe this is an appropriate frequency to separate respiratory and cardiac signals. Signals of frequencies around 50–100 kHz have been cited to exhibit the least interference from organic tissues and ambient high-frequency signals [23, 24]. Further study in an optimal carrier frequency for this application is warranted.

Any device that directly applies electrical currents to a human should be carefully evaluated in terms of both current and frequency. For a 70 kg human the minimum current of threshold perception for men and minimum threshold let-go current for women is 6 mA at 60 Hz [25]. As per the report by American national standard: *safe current limits for electromedical apparatus guidelines*; safe current limits are constant from dc to 1 kilohertz, but above one kilohertz the limit is increased proportionally to a maximum value of 100 times at 100 KHz. Therefore, at a frequency of 50 kHz the safe limit current will be 50 times the safe limit current at dc to 1 kilohertz. In our instrumentation we have limited our current to <1 mA. The voltage current intensity is so small that it cannot even be felt by the subject upon application. This amount of current will be in the safe current limit as well as high enough to be measured and analysed.

Important consideration has to be given regarding the use of an electrical impedance based gating device on patients with pacemakers or implantable cardiac defibrillator (ICD) devices. Available literature regarding the factors affecting the working of pacemakers does not specifically discuss the effect of electrical impedance devices on pacemakers. However, studies regarding similar technologies such as transcutaneous electrical nerve stimulation (TENS) have been reported. Studies have shown that TENS rarely inhibits bipolar pacing but may sometimes briefly inhibit unipolar pacing [26]. Furthermore an in-depth study of the effect of electromagnetic interference (EMI) of pacemakers [27, 28] defines one of the factors affecting EMI as the frequency of the emitting device. Frequencies between 10 kHz and 1 GHz are generally the most problematic. The signals frequencies currently employed by our device fall under this problematic range. As no substantial study has been done regarding the effect of this type of system on a pacemaker (or vice versa), the authors propose not to use the system on patients with a pacemaker or ICD until a systematic study is performed.

Selection of electrode type is also important. The adhesive Ag-AgCl disposable electrodes we have used in our study are known for their stability, reproducibility, and resistance to noise. Apart from Ag-AgCl electrodes, we have evaluated radiolucent electrodes supplied by Covidien™. We did not observe any difference in signal detection between radiolucent and Ag-AgCl electrodes. Radiolucent electrodes will be more suitable in a radiotherapy environment as these electrodes do not create any image artifacts.

The fact that the present study shows a discrepancy in the signals detected by the impedance-based and the RPM methods is most likely attributed to the detection mechanisms. Evaluation of the two methods against other well-established mechanism, such as spirometry and CT, needs to be conducted to further determine their correlations with the actual displacement of internal organs, which defines the goal of gated radiotherapy.

Electrical impedance based gating has a potential to offer advantages over conventional RPM. Firstly, the utilization of real-time impedance measurements can potentially be more accurate than guiding radiation by following the variable diaphragm motion from external blocks through a respiratory cycle. The measurement and signal processing lag from our current prototype hardware design was determined to be 33 msec or roughly 1% of a standard respiratory cycle. Such time delay is comparable with the experimentally determined RPM system time delay figures published in the earlier study [29]. It is expected that the processing required for gating of this signal will be roughly on the same order resulting in a total lag of approximately 2-3% of the standard respiratory cycle. This lag is acceptable as, according to the AAPM TG report 76, the total time delay of a real-time tracking/compensation system should be kept as short as possible and, in any case, not more than 0.5 seconds.

Placement of electrodes will be simpler than block placement for radiation therapists. The signal can be adjusted or amplified in those with only minimal chest excursion and impedance systems can capture differences in total lung volume and therefore incorporate chest expansion as well as diaphragm motion versus only the anterior/posterior motion captured in the current RPM block.

Furthermore, our study can potentially overcome the observation that paradoxical diaphragm motion (both as a single structure and with respect to the ventral rib cage) occurs in patients with emphysema [30]. As the population of lung cancer patients presenting for radiotherapy contains many patients with compromised pulmonary function, concerns about the use of the diaphragm as a surrogate indicator of lung tumor motion are extremely relevant. The electrical impedance signal is not merely based on diaphragm motion, but rather depicts changes in lung volume and geometry which has a potential to accurately gate in situations of paradoxical chest wall or diaphragm motion.

Also, as numerous reports have suggested [31–34], trans-thoracic electrical impedance can detect pulmonary fluid accumulation, the trans-thoracic bio-impedance method may be able to detect (and monitor) fluid build-up that may develop over the course of numerous radiation fractions. Fluid build-up in some circumstances may indicate a need for the treatment to be adapted or modified and would not be detected by either external marker blocks or implanted fiducials.

Importantly, while most efforts at gating have been focused on reducing lung dose and toxicity the role of cardiac gating has been largely ignored. Electrical impedance respiratory and cardiac gating in the setting of stereotactic ablative radiotherapy (SABR) for lung and esophageal treatments may allow increased dose to be delivered and increased cancer cure rates to be achieved while sparing the heart, vessels and central airways. However, the potential importance of cardiac gating is also particularly relevant for the large numbers of breast radiotherapy patients, many of whom have previously received cardiotoxic chemotherapy. As large numbers of women are treated with adjuvant breast or chest wall radiation, even small reductions in cardiac dose may be significant to survival at a population level [35–38].

The creation of a more robust, real-time, internal motion analysis system for tumor and normal tissues that integrates both respiratory and cardiac motion in a single system may allow for additional positional accuracy of critical structures and tumors affected by cardiac motion. The benefits from real-time respiratory and cardiac tracking would be paramount in SABR where long-term toxicities are often seen when high doses are given and even small errors in patient positioning or motion tracking can result in substantial overdoses to central airway or vascular structures particularly.

The utility of bio-impedance monitoring not only includes imaging at the time of treatment but could also benefit radiation planning imaging. It is now accepted that the apparent position of intrathoracic organs obtained by a free-breathing CT scan (for radiation planning) is not representative of an average position between inhalation and exhalation [39]. The use of respiratory (and potentially cardiac) gating during the CT for radiation planning would improve the positioning accuracy of tumors and normal tissues by identifying their true locations at certain phases of respiration rather than blurring their locations throughout the respiratory cycle. This would allow more accurate contouring of structures and theoretically, this technique would improve the accuracy and reproducibility of treatment.

## Conclusion

In summary, the current study indicates the feasibility of acquiring respiratory and cardiac induced bioimpedance changes simultaneously in real time using a single device. This approach has a potential for simultaneous gating during both CT simulation and radiation therapy treatment delivery. Because the current study aims to have the preliminary study of the developed system which employs the change in bioimpedance for detecting respiratory and cardiac motions, testing was only performed on a handful of human subjects. As the research in bioimpedance-based gating progresses, improvements on the system is planned, and testing on more volunteer subjects is warranted.
